# 17β-Hydroxysteroid Dehydrogenase Type 2 Inhibition: Discovery of Selective and Metabolically Stable Compounds Inhibiting Both the Human Enzyme and Its Murine Ortholog

**DOI:** 10.1371/journal.pone.0134754

**Published:** 2015-07-31

**Authors:** Emanuele M. Gargano, Giuseppe Allegretta, Enrico Perspicace, Angelo Carotti, Chris Van Koppen, Martin Frotscher, Sandrine Marchais-Oberwinkler, Rolf W. Hartmann

**Affiliations:** 1 Pharmaceutical and Medicinal Chemistry, Saarland University, Saarbrücken, Germany; 2 Helmholtz Institute for Pharmaceutical Research Saarland, Saarbrücken, Germany; 3 Dipartimento Farmaco-Chimico, Università degli Studi di Bari, Bari, Italy; 4 ElexoPharm GmbH, Saarbrücken, Germany; University of Parma, ITALY

## Abstract

Design and synthesis of a new class of inhibitors for the treatment of osteoporosis and its comparative *h*17*β*-HSD2 and *m*17*β*-HSD2 SAR study are described. **17a **is the first compound to show strong inhibition of both *h*17*β*-HSD2 and *m*17*β*-HSD2, intracellular activity, metabolic stability, selectivity toward *h*17*β*-HSD1, *m*17*β*-HSD1 and estrogen receptors *α* and *β* as well as appropriate physicochemical properties for oral bioavailability. These properties make it eligible for pre-clinical animal studies, prior to human studies.

## Introduction

Osteoporosis is a common, age-related disease, characterized by a systemic impairment of bone mass and microarchitecture, increasing bone fragility and risk of fractures [1]. It has been shown that the drop in 17β-estradiol (E2) and testosterone (T) levels, occurring with ageing, is the main factor driving the onset and progression of this disease [2]. 17β-Hydroxysteroid dehydrogenase type 2 (17*β*-HSD2) catalyzes the conversion of the highly active E2 and T into the weakly potent 17-ketosteroids estrone (E1) and Δ4-androstene-3,17-dione (Δ4-AD), respectively [3]. It is expressed in osteoblastic cells [4], therefore its inhibition can lead to the desired increase of E2 and T levels in the bone tissue and may thus be a novel approach for the treatment of osteoporosis.

Some steroidal [5–7] and non steroidal [8, 9] 17*β*-HSD2 inhibitors are already described. In our group we also developed and reported about several classes of non-steroidal 17*β*-HSD2 inhibitors [10–14], with a strong inhibition of human 17*β*-hydroxysteroid dehydrogenase type 2 (*h*17*β*-HSD2) and a good selectivity toward *h*17*β*-HSD1. Since *h*17*β*-HSD1 is the biological counterpart of *h*17*β*-HSD2, catalyzing the opposite conversion, selectivity toward this enzyme is an important feature to take into consideration. Potent and selective *h*17*β*-HSD1 inhibitors have also been described for the treatment of estrogen-dependent diseases [15, 16].

Given that the most commonly used animal model for osteoporosis studies are established in rodents [17, 18], we aimed at the development of new inhibitors displaying a good inhibition of mouse 17*β*-hydroxysteroid dehydrogenase type 2 (*m*17*β*-HSD2) and a reasonable selectivity toward *m*17*β*-HSD1. In order to have a compound suitable for animal testing and following human studies, the designed inhibitors should also display *h*17*β*-HSD2 inhibitory activity and selectivity toward *h*17*β*-HSD1. Other characteristics to be implemented are preferably low affinity to the estrogen receptors (ERs) *α* and *β* in order to maximize the E2 local effect and to minimize systemic side effects as well as metabolic stability.

Since the 3D-structure for both human and mouse 17*β*-HSD2 is up to date not available a ligand based approach was chosen for the design of new inhibitors. A set of *h*17*β*-HSD2 inhibitors was selected and tested for *m*17*β*-HSD2 inhibitory activity in order to get insight in the SAR and tracking the lead for the rational design of new inhibitors.

## Results and Discussion

*h*17*β*-HSD2, *h*17*β*-HSD1, *m*17*β*-HSD2 and *m*17*β*-HSD1 cell-free assays were performed similarly, by incubating enzyme, tritiated substrate, cofactor and inhibitor, according to described procedures [19–22].

**Fig 1:**
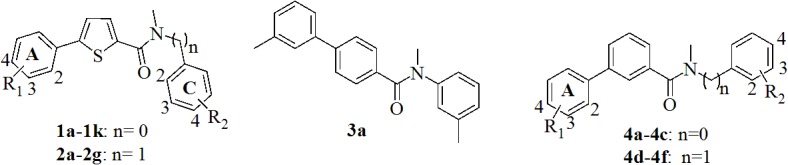
Previously described *h*17*β*-HSD2 inhibitors, tested for *m*17*β*-HSD2 inhibition.

As starting point 25 previously described *h*17*β*-HSD2 inhibitors (Fig 1), belonging to the 2,5-thiophene amide, 1,3-phenyl amide and 1,4-phenyl amide class [12–14], were tested for *m*17*β*-HSD2 inhibition, in order to elaborate a comparative SAR and to develop an optimization strategy.

IC_50_ or percent of inhibition values for both *h*17*β*-HSD2 and *m*17*β*-HSD2 are given (Table 1, compounds **1a-2g**, **3a**, **4a-4f**) to facilitate comparison.

**Table 1:** Inhibition of Human and Mouse 17*β*-HSD2 by 2,5-Thiophene Amide, 1,3-Phenyl Amide and 1,4-Phenyl Amide Derivatives in a Cell-Free Assay.

			IC_50_ (nM)^a^ or % inh. at 1 μM^a^ ^,^ ^d^	
Cmpd	R_1_	R_2_	*h*17*β*-HSD2^b^	*m*17*β*-HSD2^c^
**1a**	3-OMe	3-OMe	68	29%
**1b**	3-Me	3-Me	52	42%
**1c**	3-Me	3-OMe	58	30%
**1d**	2-OMe	3-OMe	490	19%
**1e**	3-OH	3-OH	33%	n.i.
**1f**	2-OH	3-OH	410	18%
**1g**	3-N(Me)_2_	3-OMe	170	40%
**1h**	3-F	3-OMe	510	n.i.
**1i**	4-CN	3-OMe	48%	n.i.
**1j**	2-F, 3-OMe	3-OMe	62	26%
**1k**	2-F, 3-OMe	3-Me	62	45%
**2a**	2-F, 3-OMe	3-OH	61	65%
**2b**	3-OMe	3-OMe	370	16%
**2c**	3-OH	3-OH	390	26%
**2d**	4-OH	3-OH	330	35%
**2e**	3-Me	3-OH	160	45%
**2f**	3-F	3-OH	330	37%
**2g**	4-CN	3-OH	n.i.	n.i.
**3a**	3-Me	3-Me	1100	50%
**4a**	3-OMe	3-OMe	520	25%
**4b**	4-OMe	3-OMe	1200	11%
**4c**	3-OH	3-OH	35%	n.i.
**4d**	3-OMe	3-OMe	11%	n.i.
**4e**	3-OH	3-OH	640	29%
**4f**	4-OH	3-OH	480	22%

^a^Mean value of at least two determinations, standard deviation less than 20%.

^b^Human placental microsomal fraction, substrate E2 [500 nM], cofactor NAD^+^ [1500 μM].

^c^Mouse liver microsomal fraction, substrate E2 [500 nM], cofactor NAD^+^ [1500 μM].

^d^n.i.: no inhibition, i.e., inhibition ≤10%.

In the 2,5-thiophene amide class (compounds **1a-2g**), *h*17*β*-HSD2 inhibitors [13, 14], a broad range of inhibitory activities was detected, depending on the substitution pattern; the most active compounds show IC_50_ values around 60 nM (Table 1). Conversely, the inhibitory activity towards *m*17*β*-HSD2 was only marginally affected by these changes (inhibitory activity around 30% at 1 μM). Only compound **2a** shows a more pronounced *m*17*β*-HSD2 inhibition combined with good inhibition of the human enzyme (65% at 1 μM and IC_50_ = 61 nM). Unfortunately, compound **2a** turned out to be metabolically very unstable, with a half-life of only 4 minutes in the human liver S9 fraction [13]. In a further screening, all the tested 2,5-thiophene amide displayed a high metabolic instability [13].

It is striking that neither the nature of substituents on ring A and C or their substitution pattern does appear to exert an effect on *m*17*β*-HSD2 inhibition, whereas it is decisive for the *h*17*β*-HSD2 one. This result suggests that the inhibitors in this class are likely to have different binding modes in the two enzyme isoforms.

Exchange of the central thiophene by a 1,3-disubstituted phenyl led to compounds **4a-4c** for n = 0 and **4d-4f** for n = 1, with weak inhibitory activity towards both the human and the mouse enzyme (Table 1).

**Fig 2:**
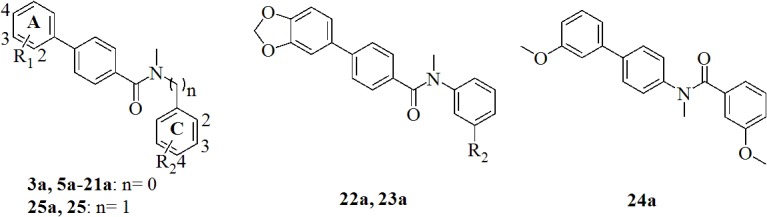
Chemical Structures of the Designed Compounds.

In contrast, compound **3a** (Table 1), bearing a 1,4-disubstituted phenyl moiety as central ring, shows moderate inhibition of both *h*17*β*-HSD2 and *m*17*β*-HSD2 and also revealed exceptional metabolic stability in the human liver S9 fraction, with a half-life time>120 minutes [13]. It was therefore taken as starting point for the design of a small library of inhibitors where the substitution pattern and the physicochemical nature of substituents on the A and C rings was varied (Fig 2). A larger number of derivatives, bearing substituents with different physicochemical properties on the A ring were prepared, according to their chemical accessibilities. Compounds **25a** and **25** were also synthesized to investigate the effect of the methylene linker between the amide function and the C ring.

**Fig 3:**
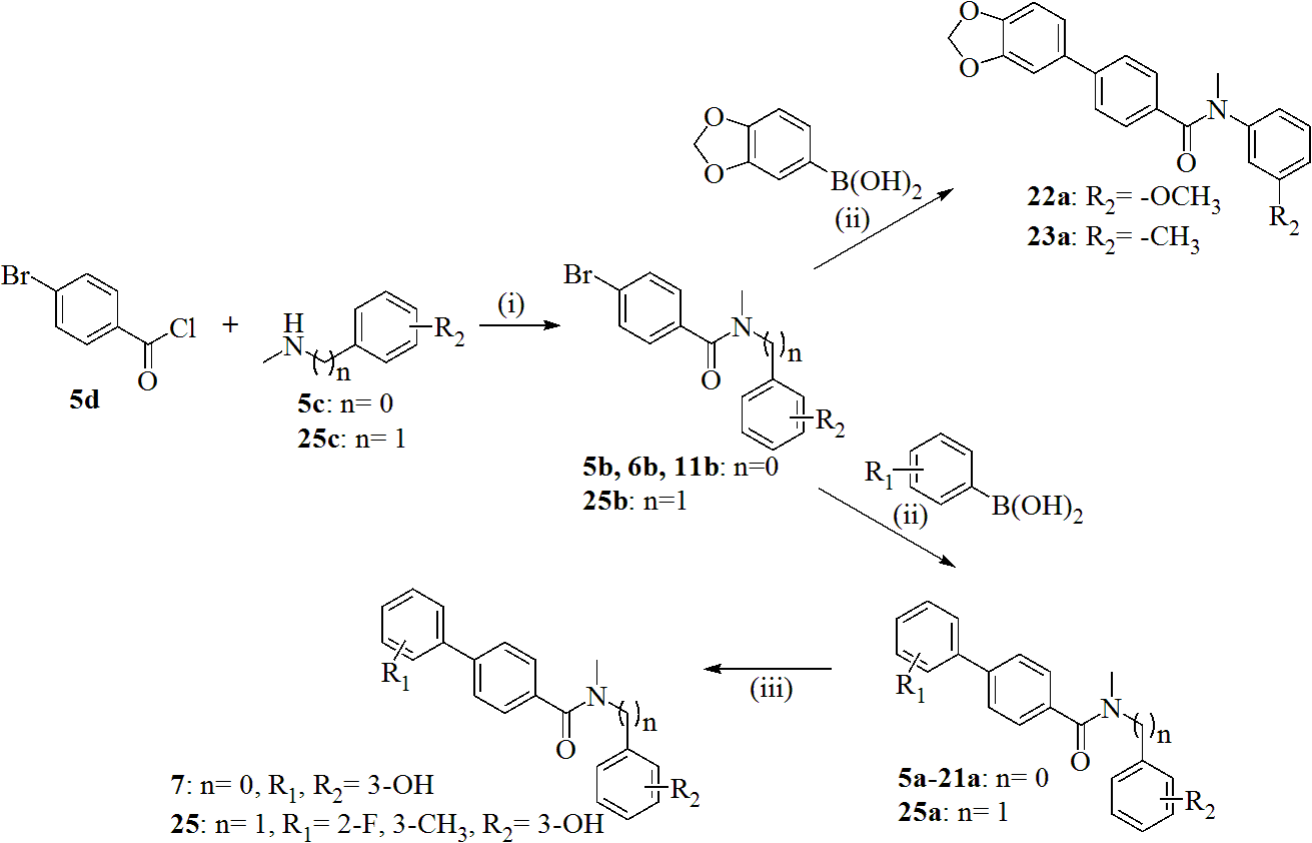
Synthesis of 1,4-Phenyl Derivatives 5a-23a, 25a, 7, 25. Reagents and conditions: (i) Et_3_N, CH_2_Cl_2_, room temperature, overnight; (ii) DME/EtOH/H_2_O (1:1:1), Cs_2_CO_3_, Pd(PPh_3_)_4_, microwave irradiation (150°C, 150W, 20 min); (iii) BF_3_·SMe_2_, CH_2_Cl_2_, room temperature, overnight.

The synthesis of the 1,4-phenyl derivatives **5a-23a, 25a, 7** and **25**, depicted in Fig 3, was accomplished following a two- or three-step reaction pathway.

First, amidation was carried out by reaction of the commercially available 4-bromobenzoyl chloride **5d** with substituted anilines **5c** or with the 1-(3-methoxyphenyl)-*N*-methylmethanamine **25c** using standard conditions (method A: triethylamine, dichloromethane, from 0C to room temperature, overnight) affording the brominated intermediates **5b, 6b** and **11b** in almost quantitative yields. Subsequently, Suzuki coupling (Method B) using tetrakis(triphenylphosphine)palladium and cesium carbonate in a mixture of DME/EtOH/H_2_O (1:1:1,3 mL) as solvent and microwave irradiation (150°C, 150 W for 20 minutes), provided the biphenyl derivatives **5a-21a** and **25a**, and the 1,3-benzodioxole derivatives **22a** and **23a** in good yields. Compounds **7a** and **25a** were submitted to ether cleavage using boron trifluoride-dimethyl sulfide complex, yielding the hydroxylated molecules **7** and **25**.

**Fig 4:**
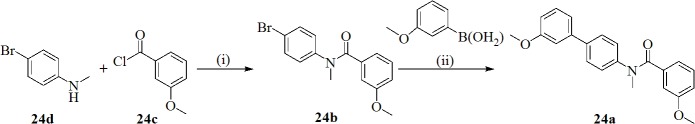
Synthesis of 1,4-Phenyl Retroamide Derivative 24a. Reagents and conditions: (i) Et_3_N, CH_2_Cl_2_, room temperature, overnight; (ii) DME/EtOH/H_2_O (1:1:1), Cs_2_CO_3_, Pd(PPh_3_)_4_, microwave irradiation (150°C, 150W, 20 min).

The synthesis of the retroamide **24a**, displayed in Fig 4, follows a two-step procedure.

First the commercially available 3-methoxybenzoyl chloride **24c** was reacted with 4-bromo-*N*-methyl aniline **24d** according to method A and afforded the brominated intermediate **24b** with 70% yield. Subsequently, Suzuki coupling following method B afforded compound **24a** in 68% yield.

Compounds **5a-25a**, **7** and **25** were tested for *h*17*β*-HSD2, *m*17*β*-HSD2 and *h*17*β*-HSD1 inhibition (Table 2, results expressed as percentage of inhibition or IC_50_ value).

**Table 2:** Inhibition of *h*17*β*-HSD2, *m*17*β*-HSD2 and *h*17*β*-HSD1 by Biphenyl Amide Derivatives with Different Substitution Patterns on the A and C Rings in Cell-Free System.

			IC_50_ (nM)^a^ or % inh. at 1 μM^a^ ^,^ ^c^			IC_50_ (nM)^a^ or % inh. at 1 μM^a^ ^,^ ^c^
Cmpd	R_1_	R_2_	*h*17*β*-HSD2^b^	*h*17*β*-HSD1^c^	s. f.^d^ ^,^ ^f^	*m*17*β*-HSD2^f^ ^,^ ^g^
**3a**	3-Me	3-Me	1100	11500	10	50%
**5a**	3-OMe	3-Me	44%	n.i.	n.d.	56%
**6a**	3-Me	3-OMe	260	6400	25	260
**7a**	3-OMe	3-OMe	330	6400	20	290
**8a**	-H	3-OMe	51%	22%	n.d.	48%
**9a**	2-OMe	3-OMe	50%	n.i.	n.d.	33%
**10a**	4-OMe	3-OMe	710	27000	38	66%
**11a**	3-OMe	4-OMe	27%	n.i.	n.d.	58%
**12a**	4-OMe	4-OMe	19%	n.i.	n.d.	62%
**7**	3-OH	3-OH	36%	15%	n.d.	42%
**13a**	3-F	3-OMe	1000	8000	8	57%
**14a**	3-Cl	3-OMe	950	12600	13	73%
**15a**	3-N(Me)_2_	3-OMe	650	5000	8	67%
**16a**	3-OMe,4-OMe	3-OMe	520	85500	164	52%
**17a**	3-Me,4-Me	3-OMe	300	13300	44	140
**18a**	3-F,4-F	3-OMe	31%	n.i.	n.d.	n.d.
**19a**	2-F, 3-OMe	3-OMe	64%	35%	n.d.	67%
**20a**	2-F, 3-Me	3-OMe	460	11300	24	48%
**21a**	2-F, 3-Me	3-Me	330	3710	11	56%
**22a**	-	3-OMe	51%	n.i.	n.d.	56%
**23a**	-	3-Me	560	10900	20	70%
**24a**	-	-	11%	n.i.	n.d.	n.d.
**25a**	2-F, 3Me	3-OMe	310	87000	283	43%
**25**	2-F, 3-Me	3-OH	260	31000	118	190

^a^Mean value of at least three determinations, standard deviation less than 20% except for **11a** (hHSD2): 26%, **7**(hHSD1): 25%,.

^b^Human placental, microsomal fraction, substrate E2 [500 nM], cofactor NAD^+^ [1500 μM].

^c^Human placental, cytosolic fraction, substrate E1 [500 nM], cofactor NADH [500 μM].

^d^s.f. (selectivity factor) = IC_50_(17*β*-HSD1)/IC_50_(17*β*-HSD2).

^e^n.i.: no inhibition (inhibition of <10%).

^f^n.d.: not determined.

^g^Mouse liver microsomal fraction, substrate E2 [500 nM], cofactor NAD^+^ [1500 μM].

Compounds **18a** and **24a** were not tested for inhibition of *m*17*β*-HSD2, due to their low inhibitory activity towards the human enzyme. Among the different synthesized 1,4-biphenyl amides without methylene linker (n = 0, Table 2, compounds **5a-21a**), the best *h*17*β*-HSD2 inhibitory activity and selectivity toward *h*17*β*-HSD1 was achieved for compounds **6a**, **7a** and **17a** (Table 2, IC_50_ values between 260 and 330nM, s.f. between 20 and 44), showing that a 3-OMe-group on ring C in combination with either a 3-OMe- or a 3-Me-group on ring A leads to a maximum in potency and selectivity in this series of compounds.

The presence of a methyl group in 4-position of ring A is tolerated by the *h*17*β*-HSD2 (compound **17a**, IC_50_ = 310nM) and increases the selectivity toward *h*17*β*-HSD1 (**17a**: s.f. 44; **6a**: s.f. 25). In contrast, compound **16a** bearing a 3,4-dimethoxy substituted A ring displays a slight decrease in *h*17*β*-HSD2 inhibitory activity if compared to the corresponding compound **7a** with only one methoxy group on that ring. The rigidification of the two methoxy substituents by the synthesis of a 1,3-benzodioxole ring (compounds **22a** and **23a)** could not overcome the drop in potency. Compounds **6a**, **7a** and **17a** displayed the strongest *m*17*β*-HSD2 inhibitory activity (IC_50_ = 260, 290 and 140 nM, respectively).

In general, the introduction of methyl- or methoxy-groups, especially in the 3-positions of rings A and B, had a positive impact on inhibitory activity, which is similar for both 17*β*-HSD2 isoforms (Table 2). Therefore, inhibitors belonging to the 1,4-phenyl class are likely to bind in a conserved area common to both enzyme, in contrast to the 2,5-thiophene amide class. As 17*β*-HSD2 belongs to the SDR superfamily, characterized by the conserved Rossmann fold and catalytic triad [23], it is possible that these inhibitors bind in or very close to these regions. Furthermore, since compounds **6a** and **17a** lack of the two oxygen functions to mimic the E2 interactions with the enzyme, they are likely to bind to the active site in an alternative mode, significantly influenced by the methyl substitution patterns.

Compounds with a methylene linker **25a** and **25** (Table 2), showed *h*17*β*-HSD2 inhibitory activity in the same order of magnitude as the corresponding derivative **20a** lacking the methylene group, but displayed improved selectivities over *h*17*β*-HSD1. In contrast to what was observed for human 17*β*-HSD2, compounds **25a** and **25** showed a significant difference in terms of *m*17*β*-HSD2 inhibition, indicating that the hydroxy group of compound **25** might function as H-bond donor in the interaction with the enzyme. A similar behavior was observed in the 2,5-thiophene amide class with regard to the *h*17*β*-HSD2 inhibitory activity[13]: all the tested 2,5-thiophene amides with a methylene linker achieved the highest *h*17*β*-HSD2 inhibition when substituted with an hydroxy group on the C ring. It might be therefore speculated, that the addition of the methylene linker can influence the binding mode of the inhibitors in both classes.

The 1,4-phenyl amides are likely to be competitive inhibitors, as derivatives with a similar structure were found to inhibit the enzyme following this mode of action.

The most potent *m*17*β*-HSD2 inhibitors **6a-8a**, **10a**, **14a**, **15a**, **17a**, **19a**, **21a**, **23a** and **25** were also tested for their *m*17*β*-HSD1 inhibitory activity. None of the tested compounds showed any inhibition of *m*17*β*-HSD1 at a concentration of 1 μM, indicating a significant selectivity toward this enzyme.

The inhibitory activity of compounds **6a**, **17a** and **25** was also evaluated in the human mammary cell line MDA-MB-231 containing endogenous 17*β*-HSD2 (Table 3). The compounds were tested at 250 nM and 1250 nM, representing approximately the IC_50_ observed in the cell-free assay, and its 5-fold value. As displayed in Table 3, all three compounds showed an inhibition between 60% and 67%at the lower concentration and approximately 90% inhibition at the higher concentration, indicating that the inhibitors can permeate the membrane and are able to inhibit the enzyme in a concentration dependent manner.

**Table 3:** Human 17*β*-HSD2 Cellular Inhibition of Compounds 6a, 17a and 25.

Cmpd	% inh^b^ HSD2^a^ at 250 nM	% inh^b^ HSD2^a^ at 1250 nM
**6a**	60%	87%
**17a**	66%	90%
**25**	67%	88%

^a^MDA-MB-231 cell line, substrate E2 [200 nM].

^b^Mean value of two determinations, standard deviation less than 15%.

Compounds **6a**, **7a**, **17a**, **21a**, **25a** and **25** were tested for their affinity toward the ERs α and β according to described methods [24] (assay details are available in the Materials and Methods). Even when applied in a 1000-fold excess relative to E2, no inhibitor was able to displace more than 20% of the steroid from the corresponding receptor, indicating a very low binding affinity to the ERs.

The metabolic stabilities of the most active compounds **6a**, **17a** and **25** were evaluated using human liver microsomes (S9 fraction). In addition, compounds **5a**, **14a**, **20a** and **25a** were also tested in order to investigate whether structure modifications might exert an effect on metabolic stability (Table 4, assay details are available in Materials and Methods). All compounds, except **25**, revealed a very high stability, which was not influenced by the nature of the substituents. Compound **25,** exhibiting a short half-life, bears a hydroxy group, potentially susceptible to phase ΙΙ metabolism. Interestingly, all the tested inhibitors from the 1,4-phenyl amide class, for n = 0, demonstrated high metabolic stability, which seemingly constitutes a positive feature of the whole class. As species differences for the metabolism of drugs that are not or only partly metabolized are usually small [25], sufficient metabolic stability in species other than human can be anticipated.

**Table 4:** Half-life in Human Liver Microsomes S9 Fraction of Representative Compounds 3a, 5a, 6a, 14a, 17a, 20a, 25a and 25.

Cmpd	R_1_	R_2_	Inhibitor^c^ t_1/2_(min)^a^ ^,^ ^b^
**3a**	3-Me	3-Me	>120
**5a**	3-OMe	3-Me	116
**6a**	3-Me	3-OMe	106
**14a**	3-Cl	3-OMe	82
**17a**	3-Me,4-Me	3-OMe	107
**20a**	2-F, 3-Me	3-OMe	104
**25a**	2-F, 3-Me	3-OMe	103
**25**	2-F, 3-Me	3-OH	6

^a^Mean of at least two determinations, standard deviation less than 25%.

^b^1 mg/ml pooled mammalian liver S9 fraction (BD Gentest), 2 mM NADPH regenerating system, 1 mM UDPGA and 0.1 mM PAPS at 37°C for 0, 5, 15 and 60 minutes.

^c^Inhibitor tested at a final concentration of 1 μM.

## Conclusion

The aim of this work was the design of a compound, which should be suitable for application in both an animal model of osteoporosis and in humans. We report here the discovery of compound **17a**, which is the first to show an appropriate profile for this purpose, with strong inhibition of both human and mouse 17*β*-HSD2 and selectivity toward the respective type 1 enzymes and the ERs. It also displayed good cellular inhibitory activity, high metabolic stability and good physicochemical parameters (MW = 345 and cLogP = 4.75) predictor for good oral bio-availability [26]. A comparative SAR study for *h*17*β*-HSD2 and *m*17*β*-HSD2 is also described for the 1,4-phenyl and the 2,5-thiophene classes of inhibitors, revealing that only compounds belonging to the first series similarly inhibit the two enzymes, probably through a similar binding mode. The species specific characterization of the thiophene and the phenyl derivatives pointed out the superiority of the latter class of inhibitors, which is able to equally inhibit the two isoenzymes and additionally displays a high metabolic stability. *In vivo* assays in a mouse osteoporosis model will be carried out soon and the results reported in due course in a specialized journal dealing with bone diseases.

## Materials and Methods

### Chemical Methods

Chemical names follow IUPAC nomenclature. Starting materials were purchased from Aldrich, Acros, Combi-Blocks or Fluorochem and were used without purification. Column chromatography was performed on silica gel (70–200 *μ*m) and reaction progress was monitored by TLC on Alugram SIL G/UV254 (Macherey-Nagel). Visualization was accomplished with UV light.^1^H NMR and ^13^C NMR spectra were measured on a Bruker AM500 spectrometer (at 500 MHz and 125 MHz, respectively) at 300 K and on Bruker Fourier 300 (at 300 MHz and 75 MHz, respectively) at 300K. Chemical shifts are reported in *δ* (parts per million: ppm), by reference to the hydrogenated residues of deuteriated solvent as internal standard: 2.05 ppm (^1^H NMR) and 29.8 and 206.3 ppm (^13^C NMR) for CD_3_COCD_3_, 7.26 ppm (^1^H NMR) and 77.0 ppm (^13^C NMR) for CDCl_3_. Signals are described as br (broad), s (singlet), d (doublet), t (triplet), dd (doublet of doublets), ddd (doublet of doublet of doublets), dt (doublet of triplets) and m (multiplet). All coupling constants (*J*) are given in Hertz (Hz).

Melting points (mp) were measured in open capillaries on a Stuart Scientific SMP3 apparatus and are uncorrected.

Mass spectrometry was performed on a TSQ Quantum (ThermoFisher, Dreieich, Germany). The triple quadrupole mass spectrometer was equipped with an electrospray interface (ESI). The Surveyor-LC-system consisted of a pump, an auto sampler, and a PDA detector. The system was operated by the standard software Xcalibur. A RP C18 NUCLEODUR 100–5 (3 mm) column (Macherey-Nagel GmbH, Dühren, Germany) was used as stationary phase. All solvents were HPLC grade. In a gradient run (acetonitrile/water) the percentage of acetonitrile (containing 0.1% trifluoroacetic acid) was increased from an initial concentration of 0% at 0 min to 100% at 15 min and kept at 100% for 5 min. The injection volume was 15 μL and flow rate was set to 800 μL/min. MS analysis was carried out at a needle voltage of 3000 V and a capillary temperature of 350°C. Mass spectra were acquired in positive mode from 100 to 1000 m/z and UV spectra were recorded at the wave length of 254 nm and in some cases at 360 nm.

All microwave irradiation experiments were carried out in a 507 CEM-Discover microwave apparatus.

All tested compounds exhibited ≥ 95% chemical purity as measured by LC/MS.

The following compounds were prepared according to previously described procedures: 4-bromo-*N*-methyl-*N*-(*m*-tolyl)benzamide **7a**[13].

#### Method A, general procedure for amide formation

To a solution of bromobenzoylchloride (2 mmol) was added the corresponding *N*-methylaniline (2 mmol) followed by Et_3_N (2 mmol) in CH_2_Cl_2_ (10 mL) at 0°C. After a few minutes, the ice bath was removed and the reaction mixture was warmed up to room temperature and stirred at room temperature overnight. The reaction mixture was extracted twice with CH_2_Cl_2_ (2 × 15 mL). The organic layer was dried over MgSO_4_, filtered and the solution was concentrated under reduced pressure. The residue was purified by silica gel column chromatography using hexanes and EtOAc as eluent or by trituration in a mixture of diethyl ether / petroleum ether to afford the desired compound.

#### Method B, general procedure for Suzuki-Miyaura coupling

In a sealed tube the previously prepared bromo-*N*-heteroarylcarboxamide derivative (1 eq.) was introduced, followed by the corresponding boronic acid (1.5 eq.), cesium carbonate (3 eq.), tetrakis(triphenylphosphine)palladium (0.02 eq.) and a mixture of DME/EtOH/H_2_O (1:1:1, v:v:v, 3 mL) as solvent. The reactor was flushed with N_2_ and submitted to microwave irradiation (150°C, 150 W) for 20 minutes. After cooling to room temperature, a mixture of EtOAc/H_2_O (1:1, v:v, 2 mL) was added to stop the reaction. The aqueous layer was extracted with EtOAc (3 × 10 mL). The organic layer was washed once with brine and once with water, dried over MgSO_4_, filtered and the solution was concentrated under reduced pressure. The residue was purified by column chromatography using hexanes and EtOAc as eluent to afford the desired compound.

### Detailed synthesis procedures of the compounds

#### 4-Bromo-*N*-(4-methoxyphenyl)-*N*-methylbenzamide (11b)

The title compound was prepared by reaction of 4-bromobenzoyl chloride (**5c**) (1272 mg, 5.8mmol) and 4-methoxy-*N*-methylaniline (**11d**) (400 mg, 2.9mmol) according to method A. The residue was purified by silica gel column chromatography (hexanes/EtOAc80:20) to afford the desired product as yellow solid (932 mg, 99%). C_15_H_14_BrNO_2_; MW 320; mp: 61–62°C; MS (ESI) 320, 322 [M]^+^; IR (cm^-1^) 1635, 2840, 2850, 2917;^1^H NMR (C_2_D_6_CO, 500 MHz) δ (ppm) 3.36 (s, 3H), 3.74 (s, 3H), 6.82 (d, *J* = 9.0 Hz, 2H), 7.09 (d, *J* = 9.0 Hz, 2H), 7.23 (d, *J* = 8.0 Hz, 2H), 7.38 (d, *J* = 8.0 Hz, 2H); ^13^C NMR (C_2_D_6_CO, 125 MHz) δ (ppm) 38.5, 55.7, 115.2, 123.7, 129.3, 131.4, 131.6, 137.1, 138.6, 159.1, 169.5.

#### 4-Bromo-*N*-(3-methoxyphenyl)-*N*-methylbenzamide (6b)

The title compound was prepared by reaction of 4-bromobenzoyl chloride(**5c**) (1272 mg, 5.8mmol) and 3-methoxy-*N*-methylaniline (**6d**) (400 mg, 2.9mmol) according to method A. The residue was purified by silica gel column chromatography (hexanes/EtOAc80:20) to afford the desired product as yellow solid (935 mg, quantitative). C_15_H_14_BrNO_2_; MW 320; mp: 98–100°C; MS (ESI) 320, 322 [M]^+^; IR (cm^-1^) 1638, 2840, 2945, 3012;^1^H NMR (C_2_D_6_CO, 500 MHz) δ (ppm) 3.40 (s, 3H), 3.71 (s, 3H), 6.70–6.72 (m, 1H), 6.74–6.78 (m, 2), 7.17 (t,*J* = 8.0 Hz, 1H), 7.26–7.28 (m, 2H), 7.39–7.41 (m, 2H); ^13^C NMR (C_2_D_6_CO, 125 MHz) δ (ppm) 38.2, 55.7, 113.2, 113.8, 120.2, 123.9, 130.7, 131.3, 131.6, 137.0, 147.0, 161.2, 169.4.

#### 4-Bromo-*N*-methyl-*N*-(*m*-tolyl)benzamide (5b)

The title compound was prepared by reaction of 4-bromobenzoyl chloride(**5c**) (724 mg, 3.3mmol) and *N*-methyl-*m*-toluidine (**5d**) (200 mg, 1.6mmol) according to method A. The residue was purified by silica gel column chromatography (hexanes/EtOAc80:20) to afford the desired product as white solid (500 mg, 99%). C_15_H_14_BrNO; MW 304; mp: 90–92C; MS (ESI) 304, 306 [M]^+^; IR (cm^-1^) 1586, 1638, 2853, 2923, 3058;^1^H NMR (C_2_D_6_CO, 300 MHz) δ (ppm) 2.24 (s, 3H), 3.39 (s, 3H), 6.90–6.93 (d, *J* = 8.0Hz, 1H), 6.99–7.05 (m, 2H), 7.14 (t, *J* = 8.0 Hz, 1H), 7.23–7.26 (m, 2H), 7.37–7.40 (m, 2H); ^13^C NMR (C_2_D_6_CO, 75 MHz) δ (ppm) 21.2, 38.4, 123.9, 125.2, 128.1, 128.5, 129.8, 131.4, 131.6, 137.0, 140.0, 145.8, 169.4.

#### 4-Bromo-*N*-(3-methoxybenzyl)-*N*-methylbenzamide (25b)

The title compound was prepared by reaction of 4-bromobenzoyl chloride (**5c**) (579 mg, 2.6mmol) and 3-methoxy-*N*-methylbenzylamine (**25d**) (200 mg, 1.3mmol) according to method A. The residue was purified by silica gel column chromatography (hexanes/EtOAc80:20) to afford the desired product as yellow oil(430 mg, 97%).C_16_H_16_BrNO_2_; MW 334; MS (ESI) 334, 336 [M]^+^; IR (cm^-1^) 1630, 2834, 2923;^1^H NMR (C_2_D_6_CO, 500 MHz) δ(ppm) 2.91 (s, 3H), 3.79 (s, 3H), 4.52–4.70 (m, 2H), 6.76–6.80 (m, 1H), 6.86 (dd, *J* = 8.8 Hz, 5Hz, 1H), 6.95 (s, 1H), 7.28 (t, *J* = 8.0 Hz, 1H), 7.44 (d, *J* = 7.6 Hz, 2H), 7.62 (s, 2H); ^13^C NMR (C_2_D_6_CO, 125 MHz) δ(ppm) 37.2, 51.0, 55.5, 113.3, 113.6, 114.4, 119.7, 121.0, 123.9, 129.9, 130.5, 132.3, 137.0, 161.1.

#### *N*-(4-bromophenyl)-3-methoxy-*N*-methylbenzamide (24b)

The title compound was prepared by reaction of 3-methoxybenzoyl chloride (**24c**) (336 mg, 2.0mmol) and 4-bromo-*N*-methylaniline(**24d**) (367 mg, 2.0mmol) according to method A. The residue was purified by silica gel column chromatography (hexanes/EtOAc80:20) to afford the desired product as white solid (440 mg, 70%). C_15_H_14_BrNO_2_; MW 320; mp: 97–100°C; MS (ESI) 320, 322 [M]^+^; IR (cm^-1^) 1634, 2834, 2929, 3009;^1^H NMR (C_2_D_6_CO, 300 MHz) δ(ppm) 3.41 (s, 3H), 3.69 (s, 3H), 6.84–6.88 (m, 3H), 7.12–7.17 (m, 3H), 7.44–7.47 (m, 2H); ^13^C NMR (C_2_D_6_CO, 75 MHz) δ(ppm) 38.3, 55.6, 114.8, 116.3, 120.1, 121.7, 129.8, 130.0, 133.1, 138.8, 145.7, 160.1, 170.3.

#### *N*-(3-methoxyphenyl)-*N*-methyl-[1,1'-biphenyl]-4-carboxamide (8a)

The title compound was prepared by reaction of 4-bromo-*N*-(3-methoxyphenyl)-*N*-methylbenzamide (**6b**)(80 mg, 0.25mmol), phenylboronic acid (40 mg, 0.33mmol), cesium carbonate (244 mg, 0.75mmol) and tetrakistriphenylphosphine palladium (0.02 eq., 6 mg) in DME/EtOH/H_2_O (1/1/1/, 3 mL) according to method B. The residue was purified by silica gel column chromatography (hexanes/EtOAc70:30) to afford the desired product as yellow solid (77 mg, 97%). C_21_H_19_NO_2_; MW 317; mp: 102–104°C; MS (ESI) 318 [M+H]^+^; IR (cm^-1^) 1588, 1636, 2831, 2917, 2960, 3040;^1^H NMR (C_2_D_6_CO, 300 MHz) δ (ppm) 3.44 (s, 3H), 3.70 (s, 3H), 6.72–6.77 (m, 2H), 6.80 (t, *J* = 2 Hz, 1H), 7.17 (t, *J* = 8 Hz, 1H), 7.33–3.37 (s, 1H), 7.41–7.46 (m, 4H), 7.51–7.54 (m, 2H), 7.60–7.63 (m, 2H); ^13^C NMR (C_2_D_6_CO, 75 MHz) δ (ppm) 38.4, 55.7, 113.0, 113.8, 120.1, 126.9, 127.7, 128.6, 129.8, 130.1, 130.6, 136.7, 140.8, 142.6, 147.4, 161.2, 170.2.

#### 2'-Methoxy-*N*-(3-methoxyphenyl)-*N*-methyl-[1,1'-biphenyl]-4-carboxamide (9a)

The title compound was prepared by reaction of 4-bromo-*N*-(3-methoxyphenyl)-*N*-methylbenzamide (**6b**) (112 mg, 0.35mmol), 2-methoxyphenylboronic acid (70 mg, 0.46mmol), cesium carbonate (342 mg, 1.05mmol) and tetrakistriphenylphosphine palladium (0.02 eq., 8 mg) according to method B. The residue was purified by silica gel column chromatography (hexanes/EtOAc70:30) to afford the desired product as colorless oil (113 mg, 93%). C_22_H_21_NO_3_; MW 347; MS (ESI) 348 [M+H]^+^; IR (cm^-1^) 1597, 1640, 2837, 2939;^1^H NMR (C_2_D_6_CO, 500 MHz) δ (ppm) 3.44 (s, 3H), 3.69 (s, 3H), 3.77 (s, 3H), 6.73–6.78 (m, 3H), 6.99 (dt, *J* = 1.0, 7.3 Hz, 1H), 7.07 (dd, *J* = 1.0, 8.3 Hz, 1H), 7.18 (dt, *J* = 1.0, 7.5 Hz, 1H), 7.24 (dd, *J* = 2.0, 7.5 Hz, 1H), 7.30–7.34 (m, 1H), 7.35–7.39 (m, 4H); ^13^C NMR (C_2_D_6_CO, 125 MHz) δ (ppm) 38.4, 55.6, 55.9, 112.5, 113.0, 113.7, 120.0, 121.7, 129.1, 129.5, 130.0, 130.4, 130.6, 131.3, 136.0, 140.7, 147.4, 157.5, 161.1, 170.4.

#### 3'-Methoxy-*N*-(3-methoxyphenyl)-*N*-methyl-[1,1'-biphenyl]-4-carboxamide (7a)

The title compound was prepared by reaction of 4-bromo-*N*-(3-methoxyphenyl)-*N*-methylbenzamide (**6b**) (81 mg, 0.25mmol), 3-methoxyphenylboronic acid (46 mg, 0.3mmol), cesium carbonate (247 mg, 0.75mmol) and tetrakistriphenylphosphine palladium (0.02 eq., 6 mg) according to method B. The residue was purified by silica gel column chromatography (hexanes/EtOAc70:30) to afford the desired product as white solid (74 mg, 85%). C_22_H_21_NO_3_; MW 347; mp: 131–134°C; MS (ESI) 348 [M+H]^+^; IR (cm^-1^) 1582, 1635, 2837, 2941, 2963;^1^H NMR (C_2_D_6_CO, 500 MHz) δ(ppm) 3.43 (s, 3H), 3.70 (s, 3H), 3.84 (s, 3H), 6.73–6.76 (m, 2H), 6.80–6.81 (m, 1H), 6.92(ddd, *J* = 1.0, 2.0, 8.0 Hz, 1H), 7.14–7.19 (m, 3H), 7.32–7.35 (m, 1H), 7.41–7.42 (m, 2H), 7.51–7.53 (m, 2H); ^13^C NMR (C_2_D_6_CO, 125 MHz) δ(ppm) 38.4, 55.6, 55.7, 113.0, 113.1, 113.8, 114.3, 120.0, 120.1, 126.9, 130.0, 130.6, 130.8, 136.8, 142.2, 142.5, 147.37, 147.38, 161.2, 170.2.

#### 3'-Hydroxy-*N*-(3-hydroxyphenyl)-*N*-methyl-[1,1'-biphenyl]-4-carboxamide (7)

To a solution of 3'-methoxy-*N*-(3-methoxyphenyl)-*N*-methyl-[1,1'-biphenyl]-4-carboxamide (**7a**) (100 mg, 0.29mmol) in CH_2_Cl_2_ (10 mL) was added trifluorobromine dimethyl sulphide complex BF_3_.SMe_2_ (0.36 mL, 3.46 mM) at room temperature. The reaction mixture was stirred overnight. The reaction was quenched with MeOH (10 mL). The solvent was removed under reduced pressure at 25°C. Water was added to dissolve inorganic salts and the precipitate was filtered off and triturated with diethyl ether to afford the desired product as white solid (80 mg, 87%). C_20_H_17_NO_3_; MW 319; mp: 168–171°C; MS (ESI) 320[M+H]^+^; IR (cm^-1^) 1589, 3191;^1^H NMR (C_2_D_6_CO, 300 MHz) δ(ppm) 3.42 (s, 3H), 6.64–6.68 (m, 3H), 6.82–6.86 (m, 1H), 7.07–7.12 (m, 3H), 7.26 (t, *J* = 8.0 Hz, 1H), 7.39–7.44 (m, 2H), 7.47–7.49 (m, 2H), 8.42 (s, 1H), 8.48 (s, 1H); ^13^C NMR (C_2_D_6_CO, 75 MHz) δ(ppm) 38.4, 114.4, 114.6, 115.0, 115.6, 119.0, 119.1, 126.8, 130.1, 130.7, 130.8, 136.6, 142.3, 142.7, 147.4, 158.8, 158.9, 170.1.

#### 4'-Methoxy-*N*-(3-methoxyphenyl)-*N*-methyl-[1,1'-biphenyl]-4-carboxamide (10a)

The title compound was prepared by reaction of 4-bromo-*N*-(3-methoxyphenyl)-*N*-methylbenzamide (**6b**) (123 mg, 0.38mmol), 4-methoxyphenylboronic acid (75 mg, 0.49mmol), cesium carbonate (371 mg, 1.14mmol) and tetrakistriphenylphosphine palladium (0.02 eq., 9 mg) according to method B. The residue was purified by silica gel column chromatography (hexanes/EtOAc80:20) to afford the desired product as white solid (70 mg, 53%). C_22_H_21_NO_3_; MW 347; mp: 128–130°C; MS (ESI) 348 [M+H]^+^; IR (cm^-1^) 1601, 1625, 2840, 2935, 2963;^1^H NMR (C_2_D_6_CO, 500 MHz) δ (ppm) 3.43 (s, 3H), 3.70 (s, 3H), 3.82(s, 3H), 6.72–6.75 (m, 2H), 6.78–6.79 (m, 1H), 6.98 (d, *J* = 2.3 Hz, 1H), 7.00 (d, *J* = 2.3 Hz, 1H), 7.15–7.18 (m, 1H), 7.37–7.40 (m, 2H), 7.46–7.48 (m, 2H), 7.55–7.58 (m,2H); ^13^C NMR (C_2_D_6_CO, 125 MHz) δ (ppm) 38.4, 55.6, 55.7, 112.9, 113.8, 115.2, 120.1, 126.3, 128.8, 130.1, 130.6, 130.1, 135.9, 142.4, 147.5, 160.7, 161.2, 170.3.

#### 3'-Fluoro-*N*-(3-methoxyphenyl)-*N*-methyl-[1,1'-biphenyl]-4-carboxamide (13a)

The title compound was prepared by reaction of 4-bromo-*N*-(3-methoxyphenyl)-*N*-methylbenzamide (**6b**) (100 mg,0.31mmol), 3-fluorophenylboronic acid (57 mg, 0.41mmol), cesium carbonate (303 mg, 0.93mmol) and tetrakistriphenylphosphine palladium (0.02 eq., 7 mg) according to method B. The residue was purified by silica gel column chromatography (hexanes/EtOAc70:30) to afford the desired product as yellow solid (91 mg, 89%). C_21_H_18_FNO_2_; MW 335; mp: 97–100°C; MS (ESI) 336 [M+H]^+^; IR (cm^-1^) 1587, 1637, 2834, 2923, 2960;^1^H NMR (C_2_D_6_CO, 300 MHz) δ (ppm) 3.44 (s, 3H), 3.71 (s, 3H), 6.73–6.77 (m, 2H), 6.80–6.81 (m, 1H), 7.09–7.21 (m, 2H), 7.36–7.50 (m, 5H), 7.55–7.57 (m, 2H); ^13^C NMR (C_2_D_6_CO, 75 MHz) δ (ppm) 38.4, 55.8, 113.1, 113.9, 114.3, 114.6, 115.2, 115.4, 120.2, 123.7, 127.1, 130.2, 130.8, 131.6, 131.8, 137.4, 141.2, 143.3, 143.4, 147.4, 161.3, 162.6, 165.8, 170.1.

#### 3'-Chloro-*N*-(3-methoxyphenyl)-*N*-methyl-[1,1'-biphenyl]-4-carboxamide (14a)

The title compound was prepared by reaction of 4-bromo-*N*-(3-methoxyphenyl)-*N*-methylbenzamide (**6b**) (80 mg, 0.25mmol), 3-chlorophenylboronic acid (52 mg, 0.33mmol), cesium carbonate (244 mg, 0.75mmol) and tetrakistriphenylphosphine palladium (0.02 eq., 6 mg) according to method B. The residue was purified by silica gel column chromatography (hexanes/EtOAc70:30) to afford the desired product as colorless oil (47 mg, 53%). C_21_H_18_ClNO_2_; MW 352; MS (ESI) 352, 354 [M]^+^; IR (cm^-1^) 1594, 1639, 2837, 2938;^1^H NMR (C_2_D_6_CO, 500 MHz) δ (ppm) 3.43 (s, 3H), 3.70 (s, 3H), 6.73–6.76 (m, 2H), 6.80–6.81(m, 1H), 7.17 (t,*J* = 8 Hz, 1H), 7.38–7.40 (m, 1H), 7.43–7.47 (m, 3H), 7.54–7.59 (m, 3H), 7.63–7.64 (m, 1H); ^13^C NMR (C_2_D_6_CO, 125 MHz) δ (ppm) 38.3, 55.7, 113.1, 113.8, 120.2, 126.3, 127.0, 127.6, 128.5, 130.1, 130.7, 131.4, 135.3, 137.4, 141.0, 142.9, 147.3, 161.2, 170.0.

#### *N*-(3-methoxyphenyl)-*N*,3'-dimethyl-[1,1'-biphenyl]-4-carboxamide (6a)

The title compound was prepared by reaction of 4-bromo-*N*-(3-methoxyphenyl)-*N*-methylbenzamide (**6b**) (77 mg, 0.24mmol), 3-methylphenylboronic acid (42 mg, 0.31mmol), cesium carbonate (235 mg, 0.72mmol) and tetrakistriphenylphosphine palladium (0.02 eq., 6 mg) according to method B. The residue was purified by silica gel column chromatography (hexanes/EtOAc70:30) to afford the desired product as colorless oil (56 mg, 71%). C_22_H_21_NO_2_; MW 331; MS (ESI) 332 [M+H]^+^; IR (cm^-1^) 1586, 1640, 2840, 2929;^1^H NMR (C_2_D_6_CO, 300 MHz) δ(ppm) 3.38 (s, 3H), 3.44 (s, 3H), 3.70 (s, 3H), 6.74–6.76 (m, 2H), 6.79–6.80 (m, 1H), 7.15–7.20 (m, 2H), 7.31 (t,*J* = 8 Hz, 1H), 7.39–7.43 (m, 4H), 7.49–7.52 (m, 2H); ^13^C NMR (C_2_D_6_CO, 75 MHz) δ(ppm) 21.5, 38.4, 55.7, 113.0, 113.9, 120.2, 124.9, 126.9, 128.5, 129.4, 129.8, 130.1, 130.7, 136.6, 137.4, 140.9, 142.9, 147.5, 161.3, 170.3.

#### 3'-(Dimethylamino)-*N*-(3-methoxyphenyl)-*N*-methyl-[1,1'-biphenyl]-4-carboxamide (15a)

The title compound was prepared by reaction of 4-bromo-*N*-(3-methoxyphenyl)-*N*-methylbenzamide (**6b**) (80 mg, 0.25mmol), 3-(dimethylamino)phenylboronic acid (54 mg, 0.33mmol), cesium carbonate (244 mg, 0.75mmol) and tetrakistriphenylphosphine palladium (0.02 eq., 6 mg) according to method B. The residue was purified by silica gel column chromatography (hexanes/EtOAc70:30) to afford the desired product as yellow oil (49 mg, 54%). C_23_H_24_N_2_O_2_; MW 360; MS (ESI) 361 [M+H]^+^; IR (cm^-1^) 1597, 1639, 2797, 2834, 2939;^1^H NMR (C_2_D_6_CO, 300 MHz) δ (ppm) 2.97 (s, 6H), 3.43 (s, 3H), 3.70 (s, 3H), 6.72–6.76 (m, 3H), 6.79 –-6.80 (m, 1H), 6.86–6.93 (m, 2H), 7.17 (t,*J* = 8.0 Hz, 1H), 7.23 (t, *J* = 8.0 Hz, 1H), 7.38–7.41 (m, 2H), 7.49–7.52 (m, 2H); ^13^C NMR (C_2_D_6_CO, 75 MHz) δ (ppm) 38.5, 40.8, 55.7, 111.8, 113.0, 113.9, 116.0, 120.2, 127.0, 130.0, 130.4, 130.7, 136.5, 141.6, 143.9, 147.5, 152.2, 161.3, 170.4.

#### *N*-(3-methoxyphenyl)-*N*,3',4'-trimethyl-[1,1'-biphenyl]-4-carboxamide (17a)

The title compound was prepared by reaction of 4-bromo-*N*-(3-methoxyphenyl)-*N*-methylbenzamide (**6b**) (100 mg, 0.31mmol), 3,4-dimethylphenylboronic acid (62 mg, 0.41mmol), cesium carbonate (303 mg, 0.93mmol) and tetrakistriphenylphosphine palladium (0.02 eq., 7 mg) according to method B. The residue was purified by silica gel column chromatography (hexanes/EtOAc70:30) to afford the desired product as yellow oil (106 mg, 99%). C_23_H_23_NO_2_; MW 345; MS (ESI) 346[M+H]^+^; IR (cm^-1^) 1599, 1640, 2840, 2914, 2942;^1^H NMR (C_2_D_6_CO, 500 MHz) δ(ppm) 2.26 (s, 3H), 2.29 (s, 3H), 3.43 (s, 3H), 3.70 (s, 3H), 6.73–6.75 (m, 2H), 6.79 –-6.80 (m, 1H), 7.15–7.19 (m, 2H), 7.32 (dd, *J* = 2.0Hz, 8.0Hz, 1H), 7.38–7.40 (m, 3H), 7.47–7.49 (m, 2H); ^13^C NMR (C_2_D_6_CO, 125 MHz) δ(ppm) 19.4, 19.8, 38.4, 55.6, 112.9, 113.8, 120.1, 125.0, 126.5, 128.8, 130.0, 130.6, 131.0, 136.2, 137.0, 137.8, 138.3, 142.8, 147.4, 161.2, 170.3.

#### 3',4'-Difluoro-*N*-(3-methoxyphenyl)-*N*-methyl-[1,1'-biphenyl]-4-carboxamide (18a)

The title compound was prepared by reaction of 4-bromo-*N*-(3-methoxyphenyl)-*N*-methylbenzamide (**6b**) (100 mg, 0.31mmol), 3,4-difluorophenylboronic acid (65 mg, 0.41mmol), cesium carbonate (303 mg, 0.93mmol) and tetrakistriphenylphosphine palladium (0.02 eq., 7 mg) according to method B. The residue was purified by silica gel column chromatography (hexanes/EtOAc70:30) to afford the desired product as yellow oil (97 mg, 88%). C_21_H_17_F_2_NO_2_; MW 353; MS (ESI) 354[M+H]^+^; IR (cm^-1^) 1599, 1639, 2837, 2948;^1^H NMR (C_2_D_6_CO, 500 MHz) δ (ppm) 3.43 (s, 3H), 3.70 (s, 3H), 6.73–6.76 (m, 2H), 6.80 –-6.81 (m, 1H), 7.17 (t, *J* = 8.0 Hz, 1H), 7.35–7.41 (m, 1H), 7.42–7.44 (m, 2H), 7.45–7.48 (m, 1H), 7.52–7.54 (m, 2H), 7.56 (dd, *J* = 2.0 Hz, 8.0 Hz, 1H), 7.60 (dd, *J* = 2.0 Hz, 8.0 Hz, 1H); ^13^C NMR (C_2_D_6_CO, 125 MHz) δ (ppm) 38.3, 55.7, 113.0, 113.8, 116.6, 116.7, 118.5, 118.7, 120.2, 124.3, 124.4, 126.9, 130.1, 130.7, 137.3, 140.3, 147.3, 161.2, 170.0.

#### 3',4'-Dimethoxy-*N*-(3-methoxyphenyl)-*N*-methyl-[1,1'-biphenyl]-4-carboxamide (16a)

The title compound was prepared by reaction of 4-bromo-*N*-(3-methoxyphenyl)-*N*-methylbenzamide (**6b**) (100 mg, 0.31mmol), 3,4-dimethoxyphenylboronic acid (75 mg, 0.41mmol), cesium carbonate (303 mg, 0.93mmol) and tetrakistriphenylphosphine palladium (0.02 eq., 7 mg) according to method B. The residue was purified by silica gel column chromatography (hexanes/EtOAc70:30) to afford the desired product as white solid (83 mg, 71%). C_23_H_23_NO_4_; MW 377; mp: 153–156°C; MS (ESI) 378[M+H]^+^; IR (cm^-1^) 1604, 1635, 2843, 2938, 2969, 3003, 3068;^1^H NMR (C_2_D_6_CO, 300 MHz) δ(ppm) 3.51 (s, 3H), 3.69 (s, 3H), 3.90 (s, 3H), 3.92 (s, 3H), 6.62–6.72 (m, 3H), 6.89–6.91 (d, *J* = 8.0 Hz, 1H), 7.04–7.17 (m, 3H), 7.38 (s, 4H); ^13^C NMR (C_2_D_6_CO, 75 MHz) δ(ppm) 38.6, 55.6, 56.1, 110.4, 111.6, 112.2, 113.0, 119.4, 119.6, 126.1, 129.4, 130.0, 133.2, 134.4, 142.2, 146.3, 149.2, 149.3, 160.2, 170.5.

#### 2'-Fluoro-3'-methoxy-*N*-(3-methoxyphenyl)-*N*-methyl-[1,1'-biphenyl]-4-carboxamide (19a)

The title compound was prepared by reaction of 4-bromo-*N*-(3-methoxyphenyl)-*N*-methylbenzamide (**6b**) (80 mg, 0.25mmol), 2-fluoro-3-methoxyphenylboronic acid (56 mg, 0.33mmol), cesium carbonate (244 mg, 0.75mmol) and tetrakistriphenylphosphine palladium (0.02 eq., 6 mg) according to method B. The residue was purified by silica gel column chromatography (hexanes/EtOAc 90:10) to afford the desired product as brown solid (43 mg, 47%). C_22_H_20_FNO_3_; MW 365; mp: 125–127°C; MS (ESI) 366[M+H]^+^; IR (cm^-1^) 1592, 1644, 2837, 2948, 2975, 3009;^1^H NMR (C_2_D_6_CO, 300 MHz) δ(ppm) 3.44 (s, 3H), 3.70 (s, 3H), 3.90 (s, 3H), 6.72–6.81 (m, 3H), 6.95–7.00 (m, 1H), 7.10–7.20 (m, 3H), 7.39 –-7.45 (m, 4H); ^13^C NMR (C_2_D_6_CO, 75 MHz) δ(ppm) 38.3, 55.7, 56.6, 113.1, 113.8, 113.9, 120.1, 122.4, 125.2, 125.3, 129.0, 129.1, 129.6, 130.6, 137.1, 137.5, 147.3, 161.2, 170.1.

#### 2'-Fluoro-*N*-(3-methoxyphenyl)-*N*,3'-dimethyl-[1,1'-biphenyl]-4-carboxamide (20a)

The title compound was prepared by reaction of 4-bromo-*N*-(3-methoxyphenyl)-*N*-methylbenzamide (**6b**) (72 mg, 0.22mmol), 2-fluoro-3-methylphenylboronic acid (45 mg, 0.29mmol), cesium carbonate (215 mg, 0.66mmol) and tetrakistriphenylphosphine palladium (0.02 eq., 5 mg) according to method B. The residue was purified by silica gel column chromatography (hexanes/EtOAc70:30) to afford the desired product as yellow solid (70 mg, 91%). C_22_H_20_FNO_2_; MW 349; mp: 91–94°C; MS (ESI) 350 [M+H]^+^; IR (cm^-1^) 1599, 1635, 2840, 2920, 2963, 3055;^1^H NMR (C_2_D_6_CO, 500 MHz) δ (ppm) 2.29 (d, *J* = 2.5 Hz, 3H), 3.44 (s, 3H), 3.70 (s, 3H), 6.73–6.77 (m, 2H), 6.79–6.80 (m, 1H), 7.13 (t, *J* = 8.0 Hz, 1H), 7.18 (t, *J* = 8.0 Hz, 1H), 7.25 (t, *J* = 8.0 Hz, 1H), 7.39–7.44 (m, 4H); ^13^C NMR (C_2_D_6_CO, 125 MHz) δ (ppm) 14.6, 14.7, 38.3, 55.7, 113.1, 113.8, 120.1, 125.0, 125.1, 126.2, 126.3, 128.6, 128.7, 129.0, 129.07, 129.09, 129.6, 130.6, 131.9, 132.0, 137.0, 137.9, 147.3, 157.9, 159.9, 170.1.

#### 4-(Benzo[d][1,3]dioxol-5-yl)-*N*-(3-methoxyphenyl)-*N*-methylbenzamide (22a)

The title compound was prepared by reaction of 4-bromo-*N*-(3-methoxyphenyl)-*N*-methylbenzamide (**6b**) (80 mg, 0.25mmol), benzo[d][1,3]dioxol-5-ylboronic acid (55 mg, 0.33mmol), cesium carbonate (244 mg, 0.75mmol) and tetrakistriphenylphosphine palladium (0.02 eq., 6 mg) according to method B. The residue was purified by silica gel column chromatography (hexanes/EtOAc70:30) to afford the desired product as yellow solid (68 mg, 76%). C_22_H_19_NO_4_; MW 361; mp: 139–141°C; MS (ESI) 362 [M+H]^+^; IR (cm^-1^) 1592, 1644, 2837, 2948, 2975, 3009;^1^H NMR (C_2_D_6_CO, 500 MHz) δ(ppm) 3.43 (s, 3H), 3.70 (s, 3H), 6.02 (s, 2H), 6.72–6.73 (m, 1H), 6.74–6.75 (m, 1H), 6.79 –-6.80 (m, 1H), 6.89–6.90 (m, 1H), 7.10–7.12 (m, 2H), 7.17 (t, *J* = 8.0 Hz, 1H), 7.37–7.39 (m, 2H), 7.44–7.46 (m, 2H); ^13^C NMR (C_2_D_6_CO, 125 MHz) δ(ppm) 38.4, 55.7, 102.3, 108.0, 109.4, 112.9, 113.8, 120.1, 121.4, 126.5, 130.0, 130.6, 135.0, 136.2, 142.4, 147.4, 148.5, 149.3 161.2, 170.2.

#### 3'-Methoxy-*N*-(4-methoxyphenyl)-*N*-methyl-[1,1'-biphenyl]-4-carboxamide (11a)

The title compound was prepared by reaction of 4-bromo-*N*-(4-methoxyphenyl)-*N*-methylbenzamide (**11b**) (100 mg, 0.31mmol), 3-methoxyphenylboronic acid (61 mg, 0.40mmol), cesium carbonate (303 mg, 0.93mmol) and tetrakistriphenylphosphine palladium (0.02 eq., 7 mg) according to method B. The residue was purified by silica gel column chromatography (hexanes/EtOAc70:30) to afford the desired product as colorlessoil (106 mg, 98%). C_22_H_21_NO_3_; MW 347; MS (ESI) 348 [M+H]^+^; IR (cm^-1^) 1635, 2840, 2935;^1^H NMR (C_2_D_6_CO, 300 MHz) δ (ppm) 3.40 (s, 3H), 3.75 (s, 3H), 3.85 (s, 3H), 6.82–6.86 (m, 2H), 6.92 (ddd, *J* = 1.0 Hz, 2.6 Hz, 8.3 Hz, 1H), 7.12–7.19 (m, 4H), 7.32–7.40 (m, 3H), 7.50–7.53 (m, 2H); ^13^C NMR (C_2_D_6_CO, 75 MHz) δ (ppm) 38.7, 55.6, 55.7, 113.1, 114.3, 115.2, 120.0, 126.9, 129.2, 130.1, 130.8, 136.9, 139.0, 142.2, 142.3, 159.0, 161.2, 170.2.

#### 4'-Methoxy-*N*-(4-methoxyphenyl)-*N*-methyl-[1,1'-biphenyl]-4-carboxamide (12a)

The title compound was prepared by reaction of 4-bromo-*N*-(4-methoxyphenyl)-*N*-methylbenzamide (**11b**) (78 mg, 0.24mmol), 4-methoxyphenylboronic acid (48 mg, 0.32mmol), cesium carbonate (235 mg, 0.72mmol) and tetrakistriphenylphosphine palladium (0.02 eq., 6 mg) according to method B. The residue was purified by silica gel column chromatography (hexanes/EtOAc 90:10) to afford the desired product as yellow solid (73 mg, 88%). C_22_H_21_NO_3_; MW 347; mp: 129–132°C; MS (ESI) 348 [M+H]^+^; IR (cm^-1^) 1635, 2840, 2932, 2957;^1^H NMR (C_2_D_6_CO, 300 MHz) δ(ppm) 3.39 (s, 3H), 3.74 (s, 3H), 3.83 (s, 3H), 6.82–6.82 (m, 2H), 6.98–7.01 (m, 2H), 7.11–7.14 (m, 2H), 7.34–7.37 (m, 2H), 7.44–7.47 (m, 2H), 7.54–7.57 (m, 2H); ^13^C NMR (C_2_D_6_CO, 75 MHz) δ(ppm) 38.7, 55.6, 55.7, 115.2, 126.2, 128.8, 129.2, 130.2, 133.0, 135.9, 139.1, 142.1, 159.0, 160.6, 170.3.

#### 3'-Methoxy-*N*-methyl-*N*-(*m*-tolyl)-[1,1'-biphenyl]-4-carboxamide (5a)

The title compound was prepared by reaction of 4-bromo-*N*-(3-methylphenyl)-*N*-methylbenzamide (**5b**) (123 mg, 0.40mmol), 3-methoxyphenylboronic acid (79 mg, 0.52mmol), cesium carbonate (393 mg, 1.20mmol) and tetrakistriphenylphosphine palladium (0.02 eq., 9 mg) according to method B. The residue was purified by silica gel column chromatography (hexanes/EtOAc70:30) to afford the desired product as yellow oil (101 mg, 77%). C_22_H_21_NO_2_; MW 331; MS (ESI) 332 [M+H]^+^; IR (cm^-1^) 1582, 1601, 1639, 2834, 2935, 3034;^1^H NMR (C_2_D_6_CO, 300 MHz) δ(ppm) 2.24 (s, 3H), 3.42 (s, 3H), 3.84 (s, 3H), 6.90–7.00 (m, 3H), 7.08 (s, 1H), 7.11–7.18 (m, 3H), 7.33 (t, *J* = 8.0 Hz, 1H), 7.38–7.41 (m, 2H), 7.49–7.52 (m, 2H); ^13^C NMR (C_2_D_6_CO, 75 MHz) δ(ppm) 21.2, 38.5, 55.6, 113.1, 114.3, 120.0, 125.1, 126.9, 127.9, 128.4, 129.8, 130.1, 130.8, 136.7, 139.9, 142.3, 142.5, 146.2, 161.2, 170.2.

#### 2'-Fluoro-*N*,3'-dimethyl-*N*-(*m*-tolyl)-[1,1'-biphenyl]-4-carboxamide (21a)

The title compound was prepared by reaction of 4-bromo-*N*-(3-methylphenyl)-*N*-methylbenzamide (**5b**) (93 mg, 0.31mmol), 2-fluoro-3-methylphenylboronic acid (62 mg, 0.40mmol), cesium carbonate (303 mg, 0.93mmol) and tetrakistriphenylphosphine palladium (0.02 eq., 7 mg) according to method B. The residue was purified by silica gel column chromatography (hexanes/EtOAc70:30) to afford the desired product as white solid (103 mg, quantitative). C_22_H_20_FNO; MW 333; mp: 73–76°C; MS (ESI) 334 [M+H]^+^; IR (cm^-1^) 1604, 1634, 2853, 2920;^1^H NMR (C_2_D_6_CO, 500 MHz) δ (ppm) 2.24 (s, 3H), 2.28 (d, *J* = 2.4 Hz, 3H), 3.43 (s, 3H), 6.94–6.97 (m, 1H), 6.98–7.01 (m, 1H), 7.07 (s, 1H), 7.11–7.16 (m, 2H), 7.23 –-7.26 (m, 2H), 7.38–7.41 (m, 4H); ^13^C NMR (C_2_D_6_CO, 125 MHz) δ (ppm) 14.6, 14.7, 21.2, 38.5, 125.05, 125.09, 125.12, 126.2, 126.3, 128.5, 128.6, 128.7, 128.99, 129.01, 129.07, 129.09, 129.6, 129.8, 131.9, 132.0, 136.9, 137.8, 139.9, 146.1, 157.9, 159.9, 170.1.

#### 4-(Benzo[d][1,3]dioxol-5-yl)-*N*-methyl-*N*-(*m*-tolyl)benzamide (22a)

The title compound was prepared by reaction of 4-bromo-*N*-(3-methylphenyl)-*N*-methylbenzamide (**5b**) (79 mg, 0.26mmol), benzo[d][1,3]dioxol-5-ylboronic acid (56 mg, 0.34mmol), cesium carbonate (254 mg, 0.78mmol) and tetrakistriphenylphosphine palladium (0.02 eq., 6 mg) according to method B. The residue was purified by silica gel column chromatography (hexanes/EtOAc70:30) to afford the desired product as yellow solid (44 mg, 49%). C_22_H_19_NO_3_; MW 345; mp: 111–113°C; MS (ESI) 346[M+H]^+^; IR (cm^-1^) 1602, 1634, 2788, 2911, 2966;^1^H NMR (C_2_D_6_CO, 500 MHz) δ (ppm) 2.24 (s, 3H), 3.42 (s, 3H), 6.02 (s, 2H), 6.89 (d, *J* = 8.0 Hz, 1H), 6.94 (d, *J* = 8.0 Hz, 1H), 6.99 (d, *J* = 8.0 Hz, 1H), 7.07–7.11 (m, 3H), 7.14 (t, *J* = 8.0 Hz, 1H), 7.35–7.37 (m, 2H), 7.42–7.44 (m, 2H); ^13^C NMR (C_2_D_6_CO, 125 MHz) δ (ppm) 21.2, 38.5, 102.3, 107.9, 109.4, 121.4, 125.1, 126.5, 127.9, 128.4, 129.8, 130.2, 135.1, 136.2, 139.9, 142.3, 146.3, 148.5, 149.3, 170.2.

#### 2'-Fluoro-*N*-(3-methoxybenzyl)-*N*,3'-dimethyl-[1,1'-biphenyl]-4-carboxamide (25a)

The title compound was prepared by reaction of 4-bromo-*N*-(3-methoxybenzyl)-*N*-methylbenzamide (**25b**) (200 mg, 0.60mmol), 2-fluoro-3-methylphenylboronic acid (120 mg, 0.78mmol), cesium carbonate (587 mg, 1.80mmol) and tetrakistriphenylphosphine palladium (0.02 eq., 14 mg) according to method B. The residue was purified by silica gel column chromatography (hexanes/EtOAc70:30) to afford the desired product as yellow oil (200 mg, 87%). C_23_H_22_FNO_2_; MW 363; MS (ESI) 364 [M+H]^+^; IR (cm^-1^) 1630, 2834, 2923, 3034;^1^H NMR (C_2_D_6_CO, 500 MHz) δ(ppm) 2.32 (s, 3H), 2.97 (s, 3H), 3.81 (s, 3H), 4.59–4.73 (m, 2H), 6.80–7.00 (m, 3H), 7.17 (t,*J* = 8 Hz, 1H), 7.27–7.32 (m, 2H), 7.33–7.36 (m, 1H), 7.57–7.59 (m, 2H), 7.62 (s, 2H); ^13^C NMR (C_2_D_6_CO, 125 MHz) δ(ppm) 14.6, 14.7, 37.3, 51.0, 55.5, 113.6, 114.4, 121.0, 125.1, 125.2, 126.2, 126.3, 128.1, 128.7, 128.8, 129.1, 129.2, 129.7, 129.8, 130.5, 132.0, 132.1, 137.1, 138.0, 158.0, 159.9, 161.1.

#### 2'-Fluoro-*N*-(3-hydroxybenzyl)-*N*,3'-dimethyl-[1,1'-biphenyl]-4-carboxamide (25)

To a solution of 2'-fluoro-*N*-(3-methoxybenzyl)-*N*,3'-dimethyl-[1,1'-biphenyl]-4-carboxamide (**25a**) (100 mg, 0.28mmol) in CH_2_Cl_2_ (10 mL) was added trifluorobromine dimethyl sulphide complex BF_3_.SMe_2_ (0.18 mL, 1.68mM) at room temperature. The reaction mixture was stirred overnight. The reaction was quenched with MeOH (10 mL). The solvent was removed under reduced pressure at 25°C. Water was added to dissolve inorganic salts and the precipitated was filtered off and triturated with diethyl ether to afford the desired product as yellow solid (66 mg, 67%). C_22_H_20_FNO_2_; MW 349; mp: 123–126°C; MS (ESI) 350 [M+H]^+^; IR (cm^-1^) 1600, 2917, 3181;^1^H NMR (C_2_D_6_CO, 500 MHz) δ (ppm) 2.32 (s, 3H), 2.96 (s, 3H), 4.55–4.69 (m, 2H), 6.72–6.90 (m, 3H), 7.16–7.22 (m, 2H), 7.29 (t, *J* = 8.0 Hz, 1H), 7.33 –-7.36 (m, 1H), 7.57–7.58 (m, 2H), 7.62 (s, 2H); ^13^C NMR (C_2_D_6_CO, 75 MHz) δ (ppm) 14.6, 14.7, 115.2, 125.1, 125.2, 126.2, 126.4, 128.0, 128.7, 128.9, 129.17, 129.21, 129.7, 129.8, 130.6, 132.0, 132.1, 137.0, 138.0, 157.3, 158.6, 160.6

#### 3-Methoxy-*N*-(3'-methoxy-[1,1'-biphenyl]-4-yl)-*N*-methylbenzamide (24a)

The title compound was prepared by reaction of *N*-(4-bromophenyl)-3-methoxy-*N*-methylbenzamide (**24b**) (80 mg, 0.25mmol), 3-methoxyphenylboronic acid (50 mg, 0.33mmol), cesium carbonate (244 mg, 0.75mmol) and tetrakistriphenylphosphine palladium (0.02 eq., 6 mg) according to method B. The residue was purified by silica gel column chromatography (hexanes/EtOAc70:30) to afford the desired product as yellow oil (59 mg, 68%). C_22_H_21_NO_3_; MW 347; MS (ESI) 348 [M+H]^+^; IR (cm^-1^) 1640, 2840, 2936;^1^H NMR (C_2_D_6_CO, 300 MHz) δ(ppm) 3.45 (s, 3H), 3.66 (s, 3H), 3.85 (s, 3H), 6.80 –-6.84 (m, 1H), 6.90–6.94 (m, 3H), 7.09–7.19 (m, 3H), 7.24–7.27 (m, 2H), 7.34 (t, *J* = 8.0 Hz, 1H), 7.57–7.60 (m, 2H); ^13^C NMR (C_2_D_6_CO, 75 MHz) δ(ppm) 38.4, 55.6, 55.7, 113.2, 114.0, 114.9, 116.4, 120.0, 121.9, 128.3, 128.4, 129.7, 130.9, 139.0, 139.6, 142.3, 145.7, 160.1, 161.3, 170.3.

### logP Determination

The logP values were calculated from ACD/Labs Percepta 2012 Release program. The logarithm of partition constant P (log P) was calculated using the “GALAS” method (Global Adjusted Locally According to Similarity). The program predicts clogP by comparing the molecule with structurally similar molecules where experimental data are known.

### Biological methods

[2,4,6,7-^3^H]-E2 and [2,4,6,7-^3^H]-E1 were purchased from Perkin-Elmer, Boston. Quickszint Flow 302 scintillator fluid was bought from Zinsser Analytic, Frankfurt. Other chemicals were purchased from Sigma, Roth or Merck.

#### Ethics statement

Anonymized placental samples were obtained from Saarbrücken-Dudweiler Hospital’s Department of Gynecology. No author involved in this study has received information about the patients. The microsomal fraction of the mouse enzyme (m17β-HSD2) was obtained from mouse livers, which were bought from Pharmacelsus GmbH (Saarbrücken, Germany).

#### *h*17*β*-HSD1 and *h*17*β*-HSD2 enzyme preparation

Cytosolic (*h*17*β*-HSD1) and microsomal (*h*17*β*-HSD2) fractions were obtained from human placenta according to previously described procedures [20, 27]. Fresh tissue was homogenized and the enzymes were separated from the mitochondria, cell membrane. Nucleus and other rests by fractional centrifugation at 1000 g, 10.000 g and 150.000 g. The pellet fraction containing the microsomal *h*17*β*-HSD2 was used for the determination of *h*17*β*-HSD2 inhibition, while *h*17*β*-HSD1 was obtained after precipitation with ammonium sulfate from the cytosolic fraction for use of testing of *h*17*β*-HSD1 inhibition. Aliquots containing *h*17*β*-HSD1 or *h*17*β*-HSD2 were stored frozen.

#### *m*17*β*-HSD2 enzyme preparation and inhibition

The microsomal fraction (*m*17*β*-HSD2) was obtained from mouse liver as described for *h*17*β*-HSD2. Inhibitory activities were evaluated by a method identical to the one described for the human enzyme.

#### *m*17*β*-HSD1 enzyme preparation

Recombinant *m*17*β*-HSD1 enzyme was produced by transfection of HEK 293 cells with a *m*17*β*-HSD1 expression plasmid (coding sequence of NM_010475 in pCMV6Entry vector, OriGene Technologies, Inc.) according to a described procedure[28]. 48 hours after transfection cells were homogenized by sonication (3 x 10 s) in a buffer containing saccharose (40 mMTris, 250 mM saccharose, 5 mM EDTA, 7 mM DTT, 1 mM PMSF, pH 7,5). Cell lysate was centrifuged (1000 g, 15 min, 4°C) and 20% glycerol was added to the supernatant before aliquots were frozen and stored at -70°C.

#### Inhibition of *h*17*β*-HSD2 and *m*17*β*-HSD2 in cell-free assay

Inhibitory activities were evaluated following an established method with minor modifications[29–31]. Briefly, the enzyme preparation was incubated with NAD^+^ [1500 μM] in the presence of potential inhibitors at 37C in a phosphate buffer (50 mM) supplemented with 20% of glycerol and EDTA 1mM. Inhibitor stock solutions were prepared in DMSO. Final concentration of DMSO was adjusted to 1% in all samples. The enzymatic reaction was started by addition of a mixture of unlabelled- and [^3^H]-E2 (final concentration: 500 nM, 0.11 μCi). After 20 min, the incubation was stopped with HgCl_2_ and the mixture was extracted with ether. After evaporation, the steroids were dissolved in acetonitrile/water (45:55). E1 and E2 were separated using acetonitrile/water (45:55) as mobile phase in a C18 RP chromatography column (Nucleodur C18, 3μm, Macherey-Nagel, Düren) connected to a HPLC-system (Agilent 1100 Series, Agilent Technologies, Waldbronn). Detection and quantification of the steroids were performed using a radioflow detector (Berthold Technologies, Bad Wildbad). The conversion rate was calculated according to the following equation: %conversion = (%E1/(%E1+%E2))×100. Each value was calculated from at least two independent experiments.

#### Inhibition of *h*17*β*-HSD1 and *m*17*β*-HSD1 in cell-free assay

The 17*β*-HSD1 inhibition assay was performed similarly to the *h*17*β*-HSD2 test. The human cytosolic enzyme was incubated with NADH [500 μM] while the mouse recombinant enzyme was reacted with NADPH [500 μM].Test compound and a mixture of unlabelled- and [^3^H]-E1 (final concentration: 500 nM, 0.15 μCi) were added and mixed for 10 min at 37°C. Further treatment of the samples and HPLC separation was carried out as mentioned above for *h*17*β*-HSD2.

#### Inhibition of *h*17*β*-HSD2 in a cellular assay

Cellular *h*17*β*-HSD2 inhibitory activity is measured using the breast cancer cell-line MDA-MB-231[32] (17*β*-HSD1 activity negligible). [^3^H]-E2 (200 nM) is taken as substrate and is incubated with the inhibitor for 6 h at 37°C. After ether extraction, substrate and product are separated by HPLC and detected with a radioflow detector. Potency is evaluated as percentage of inhibition (inhibitor concentrations used: 1250 nM and 250 nM).

#### Estrogen receptor affinity in a cell-free assay

The binding affinity of selected compounds to ER*α* and ER*β* was determined according to the recommendations of the US Environmental Protection Agency (EPA) by their Endocrine Disruptor Screening Program (EDSP)[24] using recombinant human proteins. Briefly, 1 nM of ER*α* and 4 nM of ER*β*, respectively, were incubated with [^3^H]-E2 (3 nM for ER*α* and 10 nM for ER*β*) and test compound for 16–20 h at 4°C.

The potential inhibitors were dissolved in DMSO (5% final concentration). Evaluation of non-specific-binding was performed with unlabeled E2 at concentrations 100-fold of [^3^H]-E2 (300 nM for ER*α* and 1000 nM for ER*β*). After incubation, ligand-receptor complexes were selectively bound to hydroxyapatite (83.5 g/LinTE-buffer). The bound complex was washed three times and resuspended in ethanol. For radiodetection, scintillator cocktail (Quickszint 212, Zinsser Analytic, Frankfurt) was added and samples were measured in a liquid scintillation counter (1450 LSC & Luminescence Counter, Perkin Elmer).

From these results the percentage of [^3^H]-E2 displacement by the compounds was calculated. The plot of % displacement versus compound concentration resulted in sigmoidal binding curves. The compound concentrations necessary to displace 50% of the receptor bound [^3^H]-E2 were determined. Unlabeled E2 IC_50_ values were determined in each experiment and used as reference. The E2 IC_50_ determined were 3±20% nM for *ERα* and 10±20% nM for ER*β*.

Relative Binding Affinity was determined by applying the following equation: RBA[%] = (IC_50_(E2)/IC_50_(compound)) ∙ 100[24]. This results in a RBA value of 100% for E2.

After the assay was established and validated, a modification was made to increase throughput. Compounds were tested at concentrations of 1000 times the IC_50_(E2). Compounds with less than 50% displacement of [^3^H]-E2 at a concentration of 1000 times IC_50_(E2) were classified as RBA <0.1%.

#### Metabolic Stability in a cell-free assay

Compounds **7a**, **8a**, **16a**, **19a**, **22a**, **27a** and **27** were tested according to established method[33–35]For evaluation of phase I and II metabolic stability 1 μM compound was incubated with 1 mg/ml pooled mammalian liver S9 fraction (BD Gentest), 2 mM NADPH regenerating system, 1 mM UDPGA and 0.1 mM PAPS at 37°C for 0, 5, 15 and 60 minutes at a final volume of 100 μL. The incubation was stopped by precipitation of S9 enzymes with 2 volumes of cold acetonitrile containing internal standard. Concentration of the remaining test compound at the different time points was analyzed by LC-MS/MS and used to determine half-life (t_1/2_).
